# Looking for new strategies to fight against mosquito-borne diseases: toward the development of natural extracts for mosquito control and reduction of mosquito vector competence

**DOI:** 10.3389/fphys.2013.00020

**Published:** 2013-02-13

**Authors:** Rubén Bueno-Marí

**Affiliations:** Entomology and Pest Control Laboratory, Cavanilles Institute of Biodiversity and Evolutionary Biology, University of ValenciaValencia, Spain

This commentary highlights key points, basic ideas, and future outlooks presented by Eastep et al. (2012) in *Frontiers in Physiology-Systems Biology*. The authors have provided an interesting investigation about the successful use of an environmentally friendly product derived from plants as a larvicidal agent to control mosquito populations as well as a substance that could alter the vector competence of mosquitoes for arthropod-borne viruses (arboviruses). Specifically Eastep and collaborators used coffee extracts (with and without caffeine) to try to answer two hypothesis: first, coffee extracts could have good results as a mosquitocidal compounds applied in larval biotopes and second, virus replication can be modulated in adult mosquitoes by exposing the larvae to sublethal concentrations of coffee extracts. Detailed information about results and methodology can be found in that article, but we can anticipate that both hypotheses were affirmative confirmed. Although some aspects remained unknown and must be further analyzed, e.g., the specific identification of the active agent or agents responsible for the observed effects, these preliminary results provide a new and hopeful way of fight against mosquito-borne viruses, since two flanks of the complex eco-epidemiological cycle of arbovirus can be attacked: density decrease of disease vectors and reduction/inhibition of the virus replication mosquitoes. However, the influence of coffee extracts exposure on vectorial capacity (VC) of mosquitoes must be carefully analyzed, since VC depends of a great variety of parameters such as vector density, host feeding preference, number of gonotrophic cycles, adult survival, or extrinsic incubation period, among others. Despite Eastep and colleagues detected high mortality in mosquito larvae exposed to coffee extracts, it must be noted that the same authors also evidenced that females reared in coffee tended to lay more eggs than control females, which could theoretically lead to a higher risk of virus transmission by increasing the vector density at medium or long term. Therefore, possible adverse implications of larval biotopes alterations by applying coffee extracts should be deeply and meticulously studied.

It is interesting to note that mosquito species selected to carry out the assays was *Aedes albopictus* (Skuse, 1894), which is currently one of the most important threats for public health all over the world due to its invasive behavior and potential to transmit a great broad of diseases including some devastating viruses for human population such as Dengue, Yellow Fever, or Chikungunya (Bueno Marí and Jiménez Peydró, 2012). However, none of these tropical and anthroponotic viruses were selected to test the antiviral experiments. The virus employed was La Crosse virus (LACV), which is a zoonotic virus of Bunyaviridae family that usually involves chipmunks and accidentally humans, where infections are common in non-tropical countries like United States of America (USA) but disease is not frequent and symptoms are generally of low severity causing occasionally encephalitis and very rarely death (Borucki et al., 2002; Haddow and Odoi, 2009). Nevertheless, some references about antiviral activity of plant extracts against other mosquito-borne diseases such as Dengue (Tang et al., 2012), Yellow Fever (Ojo et al., 2009), or Ross River Fever (Semple et al., 1998), among others, can be found in literature.

The paper of Eastep and collaborators affects to an important issue nowadays for public institutions and private companies responsible for mosquito control campaigns: the development of natural insecticides with scanty resistance effects and low toxicity to humans and non-target organisms of aquatic environments. Mosquito resistance is currently an increasing problem due to the almost exclusively use of chemical insecticides to manage mosquito pests in many territories during decades. Resistance may develop due to changes in enzyme systems of mosquitoes, resulting in more rapid detoxification or sequestration of the insecticide, or even due to mutations in the target site preventing the insecticide-target interaction (Hemingway et al., 2004). In recent years, the use of toxins derived from Bacillus species bacteria has been deeply established as one of the best tools to reduce mosquito larval populations. Although in some phenomena of resistance on mosquitoes have been described in products derived from *Bacillus* under special conditions (Paris et al., 2011), these issues must be further analyzed because there are hundreds of researches that never have been able to demonstrate the existence of resistance processes in these bacterial larvicides. In any case, to try to minimize possible appearance of resistance problems, major emphasis must be applied on the use of natural plant-based products as larvicides which can be a safe alternate to chemical or bacterial insecticides. At respect, more than 2000 plants species have been known to produce chemical factors and metabolites of value in pest control programs, and among these plants, products of some 344 species have been reported to have a variety of activities against mosquitoes (Sukumar et al., 1991). Unfortunately, very few plant products have been developed for controlling mosquitoes, despite some phytochemicals are able to act against mosquitoes as growth regulators, repellents, and ovipositional deterrent (Amer and Mehlhorn, 2006; Rajkumar and Jebanesan, 2007).

Finally I would like to acknowledge Eastep and colleagues for his valuable contribution to classical strategies of fight against mosquito-borne diseases and also highlight that manuscript of Eastep et al. (2012) is one of the articles selected in the Research Topic titled “*Global change and human vulnerability to vector-borne diseases*” published by *Frontiers in Physiology-Systems Biology* which includes many researches related with the use of other natural larvicides against mosquitoes (Kumar et al., 2012) or the impact of climate trends on vector-borne pathogen transmission (Estrada-Peña et al., 2012), among other themes. This Research Topic could be visited through next link:

http://www.frontiersin.org/Systems_Biology/researchtopics/GLOBAL_CHANGE_AND_HUMAN_VULNER/323.
